# Associations Between Previously Identified Genetic Variants and Clinical Phenotypes of Diabetic Neuropathy in Type 2 Diabetes: An Exploratory Analysis of the Discovery Cohort

**DOI:** 10.3390/ijms27125487

**Published:** 2026-06-17

**Authors:** Noémi Hajdú, Zsófia Ludvig, Ramóna Rácz, Ildikó Istenes, Magdolna Békeffy, Orsolya Erzsébet Vági, Anna Erzsébet Körei, Eszter Horváth, Bálint Tóbiás, Anett Illés, Henriett Pikó, János P. Kósa, Kristóf Árvai, Máté Posta, Péter András Lakatos, Péter Kempler, Zsuzsanna Putz, Dóra Zsuzsanna Tordai

**Affiliations:** 1 Department of Internal Medicine and Oncology, Semmelweis University, 1083 Budapest, Hungary; zsofiludvig@gmail.com (Z.L.); racz.ramona@semmelweis.hu (R.R.); istildi78@gmail.com (I.I.); bekeffy.magdi95@gmail.com (M.B.); vagiorsi@gmail.com (O.E.V.); korei.anna@semmelweis.hu (A.E.K.); g.horvath.eszter@semmelweis.hu (E.H.); balint.tobias@gmail.com (B.T.); illes.anett@semmelweis.hu (A.I.); piko.henriett@semmelweis.hu (H.P.); kosa.janos@semmelweis.hu (J.P.K.); lakatos.peter@semmelweis.hu (P.A.L.); kempler.peter@semmelweis.hu (P.K.); putz.zsuzsanna@semmelweis.hu (Z.P.); tordai.dora@semmelweis.hu (D.Z.T.); 2 Hungarian Research Network HUN-REN-ENDOMOLPAT, 1085 Budapest, Hungary; krisof.arvai@gmail.com; 3 Department of Pathology and Experimental Cancer Research, Semmelweis University, 1085 Budapest, Hungary; 4 Department of Bioinformatics, Semmelweis University, 1094 Budapest, Hungary; posta.mate@ttk.hu; 5 Institute of Molecular Life Sciences, HUN-REN Research Centre for Natural Sciences, 1117 Budapest, Hungary

**Keywords:** type 2 diabetes, diabetic neuropathy, cardiovascular autonomic neuropathy, sensory neuropathy, whole-exome sequencing, single-nucleotide polymorphisms, case–control, clinical associations with genetic variants

## Abstract

Diabetic neuropathy is a common and multifactorial complication of type 2 diabetes, in which genetic susceptibility is increasingly recognized as a contributing factor. This study (cross-sectional case–control) aimed to investigate the associations between previously identified genetic variants and clinically relevant neurophysiological and symptomatic parameters. A total of 48 individuals with type 2 diabetes (24 with neuropathy and 24 without) were included. Neuropathy was assessed using standardized neurological, sensory, and cardiovascular autonomic function tests. Genetic variants were selected in a prior discovery analysis of this same cohort and re-tested here, precluding independent validation. Associations between genetic variants and clinical parameters were assessed through group-based comparisons using Mann–Whitney U tests and Fisher’s exact test, correlation analysis using Spearman’s rank correlation, permutation-based testing to improve robustness, and multivariable linear regression adjusted for age and sex to account for potential demographic confounding (q < 0.1). Mann–Whitney U test analysis identified several associations between genetic variants and neuropathy-related clinical parameters. In the Mann–Whitney U test analysis, only the rs6682221 variant remained significantly associated with heat detection threshold in the left hand after false discovery rate correction (*p* = 0.000150; q = 0.02736), although this association did not remain significant in the complementary permutation analysis based on median differences. Spearman’s rank correlation analysis identified a significant positive association between rs6682221 allele burden and heat detection threshold in the left hand, which remained significant after permutation correction (r = 0.552, *p* = 0.000086, q = 0.016). Multivariable regression adjusted for age and sex revealed several independent associations between selected variants and sensory neuropathy-related parameters. These findings should be considered exploratory, as all analyses were performed within the original discovery cohort and no independent validation cohort was available. Independent replication and functional studies are required before any clinical relevance can be inferred.

## 1. Introduction

Diabetic neuropathy is one of the most common and clinically significant microvascular complications of diabetes, affecting approximately one-third to one-half of patients and substantially contributing to increased morbidity and mortality [1,2]. It is a major predictor of adverse outcomes, including diabetic foot ulcers, cardiovascular morbidity, and mortality [3,4,5]. Distal symmetric polyneuropathy (DSPN) represents the most prevalent form and is associated with reduced quality of life due to an increased risk of ulceration, amputation, and falls [6,7]. In addition, cardiovascular autonomic neuropathy (CAN) is a clinically important manifestation characterized by impaired autonomic regulation, which is associated with increased cardiovascular and all-cause mortality [8].

Despite advances in clinical management, including patient education, screening strategies, and optimization of metabolic control, the prevention and progression of diabetic neuropathy remain challenging [9]. Although improved glycemic control reduces the risk of complications, evidence suggests that its effect on microvascular outcomes is limited, indicating that additional pathogenic mechanisms contribute to disease development [2,10]. The pathogenesis of diabetic neuropathy is therefore considered multifactorial, involving complex interactions among metabolic, vascular, inflammatory, and neurodegenerative processes.

Genetic susceptibility is increasingly recognized as a factor influencing both the onset and the progression of neuropathy. However, our current knowledge regarding the contribution of genetic factors remains incomplete [11,12,13]. Recent advances in high-throughput sequencing technologies have enabled the identification of genetic variants that may modulate key biological pathways relevant to neuropathy, including neuronal survival, intracellular signaling, vascular homeostasis, and inflammatory regulation.

In our previous study, whole-exome sequencing (WES) in patients with type 2 diabetes identified several variants associated with altered neuropathy risk, including polymorphisms in the *RecQ-mediated genome instability protein 2* (*RMI2*), *myosin-binding protein H-like* (*MYBPHL*), *multivesicular body subunit 12B* (*MVB12B*), and *retinoic acid X receptor alpha* (*RXRA*) genes [14].

After further analysis of the whole-exome sequencing data, we identified the following single-nucleotide polymorphisms (SNPs) [15]. Variants in the *titin* (*TTN*) gene (rs922984, rs2291313/rs4471922) may influence cardiac muscle structure and insulin-related signaling, potentially contributing to diastolic dysfunction [16,17,18]. The rs6086563 polymorphism in the *phospholipase C-beta 1* (*PLCB1*) gene may contribute to neuronal signaling alterations through phosphoinositide and inositol metabolism pathways involved in neurotransmission, insulin resistance, nerve conduction, and microvascular dysfunction [19,20,21,22]. The intronic rs4241602 variant in the *cyclin I* (*CCNI*) gene may influence neuronal survival through cyclin-dependent kinase 5 (CDK5)-mediated regulation of apoptosis, neuronal signaling, and pain-related pathways [23,24,25]. Furthermore, intronic variants in the *cell division cycle 34* (*CDC34*) gene (rs2396295, rs892204) may affect ubiquitin-mediated protein degradation, cellular stress responses, and neuronal homeostasis, potentially contributing to neuropathy-related dysfunction [26,27,28]. Finally, the rs6682221 variant located upstream of the *BTG anti-proliferation factor 2* (*BTG2*) gene may be involved in neuronal growth, differentiation, and survival pathways [29,30].

Building on these findings, the present study aimed to explore the relationships between these gene variants and clinically relevant neurophysiological and laboratory parameters within the same patient cohort in which the variants were originally identified. As these genotype–phenotype associations were not validated in an independent cohort, the present analysis should be considered exploratory and hypothesis-generating in nature.

## 2. Results

Table 1 shows the clinical characteristics of the two study groups. While those without neuropathy were older than those with neuropathy, the groups did not differ significantly in terms of diabetes duration, Body Mass Index (BMI), HbA1c levels or lipid metabolism.

The whole-exome sequencing analysis results are summarized in Table 2 [15].

We identified eight genetic variants, representing six independent association signals due to linkage disequilibrium between selected SNP pairs, that may influence susceptibility to diabetic neuropathy [15].

Given that age and diabetes duration differed between patients with and without neuropathy, Firth logistic regression models were constructed for each investigated SNP, using neuropathy status as the dependent variable, and adjusting for sex, age, and diabetes duration. Notably, all previously identified SNP associations remained statistically significant after adjustment, suggesting that these polymorphisms retain independent additive association value within the corrected models (Supplementary Table S1). 

Elevated odds of neuropathy were observed for several variants identified in this cohort, including the rs922984 and rs2291313/rs4471922 variants in the *titin* gene (*TTN*); the rs6086563 SNP in the *phospholipase C-beta 1* gene (*PLCB1*); the rs4241602 variant in the *cyclin I* gene (*CCNI*); and rs2396295/rs892204 in the *cell division cycle 34* gene (*CDC34*). An additional finding was that the rs6682221 variant of the *BTG anti-proliferation factor 2* (*BTG2*) gene was associated with a lower estimated odds of neuropathy (OR = 0.045). Table 2 demonstrates the chromosomal locations and positions of these genetic variants, alongside their corresponding odds ratios (ORs) and 95% confidence intervals (CIs), which estimate the risk of developing neuropathy. However, the estimated odds ratios should be interpreted with caution, as the wide confidence intervals indicate limited precision and potential instability of the estimates, most likely due to the small sample size and the low number of individuals in certain genotype groups.

Pairwise linkage disequilibrium (LD) analysis demonstrated that rs2291313 and rs4471922 within the *TTN* gene are in complete LD in the European reference population (r^2^ = 1.00, D′ = 1.00), indicating that they represent the same underlying association signal rather than independent variants. Similarly, rs2396295 and rs892204 within the *CDC34* gene showed near-complete LD (r^2^ = 0.985, D′ = 1.00), supporting the interpretation that these variants reflect a shared haplotypic signal rather than separate genetic associations.

Table 3 shows the correlations between genetic variants and neurophysiological parameters with Mann–Whitney U test (to distinguish differences in clinical outcomes based on genotype).

**Figure 1:**
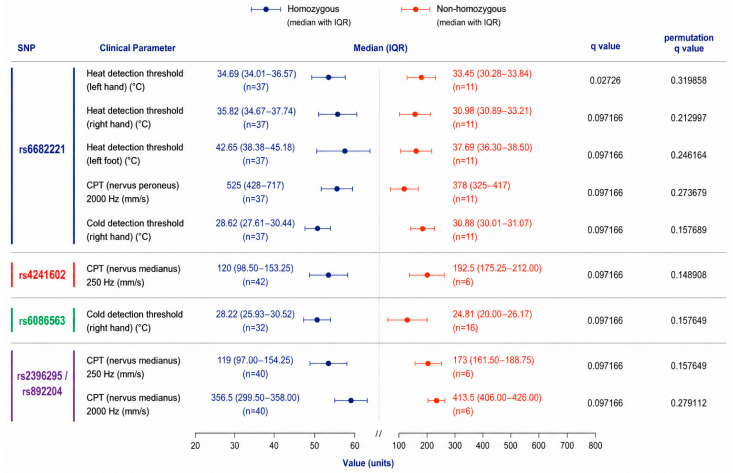
Genotype–phenotype associations in neuropathy-associated clinical and neurophysiological parameters. Data are reported as median [IQR].

Mann–Whitney U test analysis identified several associations between genetic variants and clinical parameters (Table 3, Figure 1). However, when applying multiple testing correction, only associations with q-values below 0.05 were considered statistically significant, whereas findings with q-values between 0.05 and 0.1 were interpreted as borderline and exploratory.

Among all evaluated associations in the Mann–Whitney U test analysis, only the rs6682221 variant remained statistically significant after false discovery rate (FDR) correction (q = 0.02736). However, this association did not remain significant following the complementary permutation analysis based on median differences (permutation q = 0.319858).

Several additional associations met the exploratory threshold of q < 0.1. For rs6682221, exploratory associations were observed for heat detection threshold in the right hand (*p* = 0.005115; q = 0.097166) and left foot (*p* = 0.005780; q = 0.097166), current perception threshold at 2000 Hz for nervus peroneus (*p* = 0.002849; q = 0.097166) and cold detection threshold in the right hand (*p* = 0.005551; q = 0.097166). The rs4241602 variant demonstrated an exploratory association with current perception threshold at 250 Hz for nervus medianus (*p =* 0.002134; q = 0.097166). Similarly, rs6086563 showed an exploratory relationship with cold detection threshold in the right hand (*p* = 0.004122; q = 0.097166). The rs2396295/rs892204 variants demonstrated exploratory associations with current perception threshold at 250 Hz for nervus medianus (*p* = 0.006162; q = 0.097166) and current perception threshold at 2000 Hz for nervus medianus (*p* = 0.006413; q = 0.097166).

After permutation correction, none of the observed associations retained statistical significance, indicating that these findings may be sensitive to sample size limitations and multiple-testing burden. Therefore, the reported associations should be interpreted cautiously and regarded as exploratory and hypothesis-generating rather than confirmatory.

Fisher’s exact test (Table 4) revealed exploratory associations between Neuropathy Total Symptom Score-6 (NTSS-6) and the investigated genetic variants (q < 0.1). Specifically, rs2291313/rs4471922 showed identical results (OR = 7.14, *p* = 0.002949, q = 0.097166), while rs6086563 also showed an exploratory association (OR = 7.14, *p* = 0.004606, q = 0.097166). The elevated odds ratios indicate that individuals with the homozygous genotype have a higher likelihood of increased NTSS-6 scores, reflecting a greater burden of neuropathic symptoms.

**Figure 2:**
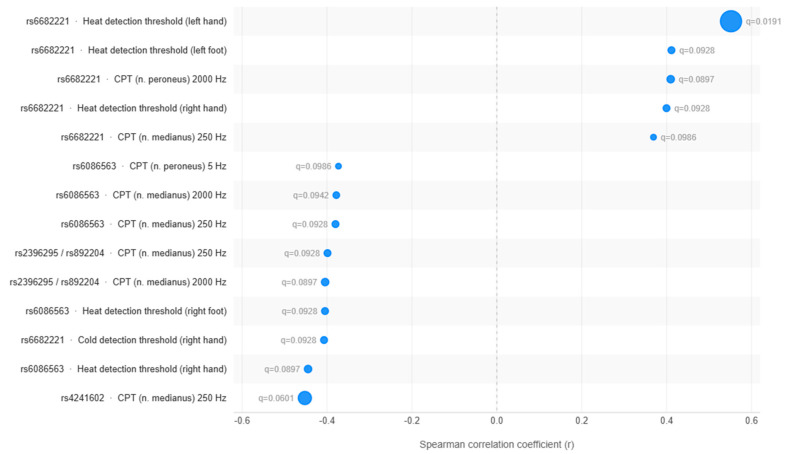
Relationship between allele dosage and neuropathy-associated measurements identified by Spearman’s rank correlation analysis with permutation correction. The plot illustrates genotype–phenotype associations with q-values < 0.1. The x-axis represents Spearman’s rank correlation coefficients (r), indicating the direction and magnitude of the association between allele dosage and clinical parameters. Bubble size reflects statistical robustness based on permutation-adjusted q-values, with larger bubbles corresponding to lower q-values. Positive correlations indicate increasing parameter values with higher allele dosage, whereas negative correlations indicate inverse associations.

Spearman’s rank correlation analysis was performed to evaluate associations between allele burden and clinical parameters (Table 5, Figure 2). Correlation coefficients, *p*-values, q-values, and permutation-adjusted q-values were calculated to assess the robustness of the observed relationships. Associations with q-values below 0.05 were considered statistically significant, whereas findings with q-values between 0.05 and 0.1 were interpreted as exploratory and borderline.

Among all tested associations, only one correlation remained statistically significant after false discovery rate correction. The rs6682221 variant demonstrated a strong positive correlation with heat detection threshold in the left hand (r = 0.552, *p* = 0.000086, q = 0.016), indicating that higher allele burden was associated with increased threshold values. Importantly, this association also remained significant after permutation-based analysis (permutation q = 0.019), supporting its relative robustness.

Several additional correlations met the exploratory threshold of q < 0.1. For rs6682221, moderate positive associations were observed with heat detection threshold in the left foot (r = 0.411, *p* = 0.0050, q = 0.079), heat detection threshold in the right hand (r = 0.400, *p* = 0.0065, q = 0.079), and current perception threshold at nervus peroneus 2000 Hz (r = 0.409, *p* = 0.0039, q = 0.079). In contrast, rs6682221 demonstrated a moderate negative correlation with cold detection threshold in the right hand (r = −0.407, *p* = 0.0055, q = 0.079), as well as a weaker positive association with current perception threshold at nervus medianus 250 Hz (r = 0.369, *p* = 0.0099, q = 0.090).

The rs892204/rs2396295 variant demonstrated moderate negative correlations with current perception threshold at nervus medianus 2000 Hz (r = −0.404, *p* = 0.0044, q = 0.079) and 250 Hz (r = −0.399, *p* = 0.0050, q = 0.079).

The rs4241602 variant demonstrated a moderate negative correlation with current perception threshold at nervus medianus 250 Hz (r = −0.452, *p* = 0.0013, q = 0.079). For rs6086563, several exploratory negative correlations were observed, including current perception threshold at nervus medianus 2000 Hz (r = −0.378, *p* = 0.0081, q = 0.086) and 250 Hz (r = −0.380, *p* = 0.0077, q = 0.086), nervus peroneus 5 Hz (r = −0.373, *p* = 0.0090, q = 0.090), cold perception threshold in the right hand (r = −0.445, *p* = 0.0022, q = 0.079), and cold perception threshold in the right foot (r = −0.405, *p* = 0.0064, q = 0.079).

Permutation-based analysis was subsequently performed to evaluate the stability of the observed correlations. Following permutation correction, only the association between rs6682221 and heat detection threshold in the left hand remained statistically significant (permutation q = 0.019). All remaining associations lost significance after permutation adjustment, indicating that they should be interpreted cautiously and considered exploratory in nature.

After adjustment for age and sex in the multivariable linear regression analysis, several associations remained statistically significant. Significant associations were observed for rs6682221 with heat detection threshold in the left hand (*p* = 0.029) and left foot (*p* = 0.011), rs4241602 with current perception threshold at nervus medianus 250 Hz (*p* = 0.002), rs6086563 with heat detection threshold in the right hand (*p* = 0.005), and rs2396295/rs892204 with current perception threshold at nervus medianus 250 Hz (*p* = 0.020) and 2000 Hz (*p* = 0.002).

## 3. Discussion

### 3.1. Summary of Findings

In our previous study [15], we identified eight genetic variants potentially associated with the development of diabetic neuropathy. The previously described genetic variants had not been reported in the literature prior to our study. Although several studies have described the corresponding genes and their potential roles in neuronal survival, apoptosis, and related biological pathways, their specific contribution to diabetic neuropathy remains unclear. Therefore, no definitive conclusions can be drawn regarding how these genetic variants may directly influence the development or progression of neuropathy.

The objective of the present study was to further investigate the relationship between these variants and clinically relevant phenotypic parameters within the same cohort. To address this aim, we applied complementary statistical approaches, including group-based comparisons using Mann–Whitney U tests and Fisher’s exact test, correlation analyses using Spearman’s rank correlation, permutation-based testing to improve robustness, and multivariable linear regression adjusted for age and sex to account for potential demographic confounding.

In the group-based comparison (Mann–Whitney U test), several genotype–phenotype associations were initially identified, including an association between rs6682221 and heat detection threshold values in the left hand that remained significant after false discovery rate correction (*p* = 0.000150; q = 0.02736). However, following permutation testing, all previously observed associations lost statistical significance, suggesting limited robustness of these findings. Therefore, no association identified in the Mann–Whitney U analysis remained significant after both FDR correction and the complementary permutation analysis based on median differences. Therefore, the results of the group-based comparison should be interpreted cautiously and considered exploratory and hypothesis-generating rather than confirmatory.

Analysis of binary variables using Fisher’s exact test revealed a borderline association between NTSS-6 scores and the investigated genetic variants (rs2291313/rs4471922, rs6086563).

In the Spearman’s rank correlation analysis, the association between rs6682221 and heat detection threshold in the left hand was the only finding that remained statistically significant after both false discovery rate correction and permutation testing. The rs6682221 variant, located upstream of the *BTG2* gene, has previously been linked to biological pathways involved in neuronal growth, differentiation, and regeneration [29,30]. Although BTG2 has been primarily studied in the context of central nervous system development, its involvement in pathways regulating neuronal survival and growth raises the possibility that similar mechanisms may also be relevant to peripheral nerve function.

Heat detection threshold reflects small-fiber sensory function and is considered a sensitive marker of early peripheral nerve dysfunction in diabetic neuropathy. Higher threshold values indicate impaired thermal perception and more pronounced small-fiber sensory impairment. Therefore, the observed positive correlation suggests that increasing rs6682221 allele dosage is associated with worsening thermal sensory function. Because this association remained significant after both false discovery rate correction and permutation testing, it represents the most robust genotype–phenotype relationship identified in the present study.

The direction of the observed correlation was unexpected, as a negative association would have been anticipated based on the presumed protective role of rs6682221 in our previous study. Several factors may explain this discrepancy. First, the original analysis investigated susceptibility to diabetic neuropathy, whereas the present study examined a quantitative measure of sensory function. Consequently, the relationship between a genetic variant and overall neuropathy susceptibility may not necessarily correspond to its association with a specific sensory phenotype. Second, the presumed protective role of rs6682221 was inferred from the findings of our previous association study rather than from direct functional evidence. Therefore, the biological effect of this variant may be more complex than initially assumed, and its relationship with specific sensory phenotypes may not necessarily follow the direction predicted from the original neuropathy susceptibility analysis. Third, the functional consequences of rs6682221 remain unknown, limiting the biological interpretation of the observed association. In addition, the observed relationship may have been influenced by the limited sample size and genotype distribution within the cohort. Because the number of wild-type genotype carriers was too limited to allow statistically meaningful subgroup evaluation as a separate category, an additive genotype coding approach was applied. This methodological constraint may have affected the estimated direction and magnitude of the observed association. Furthermore, the association was observed for a single sensory parameter, namely heat detection threshold in the left hand, and was not consistently replicated across other thermal or sensory measures. This lack of consistency warrants cautious interpretation and highlights the need for replication in independent cohorts.

Consequently, although the association between rs6682221 and heat detection threshold remained significant after stringent statistical correction, its biological and clinical significance remains uncertain. Further studies in larger, independent cohorts, as well as functional investigations of rs6682221, are required to clarify the role of this variant in diabetic neuropathy and sensory dysfunction.

After adjustment for age and sex, several associations remained statistically significant in multivariable regression models, suggesting that these genotype–phenotype relationships were not fully explained by demographic confounding. Significant associations remained for rs6682221 with heat detection threshold in the left hand and left foot, rs4241602 with current perception threshold at 250 Hz in the nervus medianus, and rs2396295/rs892204 with current perception threshold values at both 250 Hz and 2000 Hz in the nervus medianus. Notably, the association between rs6086563 and heat detection threshold in the right hand was not statistically significant in the Mann–Whitney U test but became significant after adjustment for age and sex in the multivariable regression analysis. These findings suggest that the observed associations may reflect independent effects of the investigated variants on sensory neuropathy-related phenotypes.

The Mann–Whitney U test and multivariable regression analyses address related but distinct statistical questions. Whereas the Mann–Whitney U test evaluates unadjusted differences between genotype groups, multivariable regression assesses whether genotype–phenotype associations remain independent of demographic covariates. Consequently, discrepancies between these approaches may reflect confounding by demographic variables, differences in statistical power, or the different assumptions underlying the analytical methods. Because the regression analyses were performed as exploratory secondary analyses and were not subjected to the same permutation-based significance assessment as the primary analyses, their results should be interpreted as supportive rather than confirmatory evidence.

The partially inconsistent and, in some cases, contradictory findings observed in the present study may be explained by the limitations outlined above. Consequently, the observed associations should be interpreted with caution. Rather than providing confirmatory evidence, this study should be regarded as an exploratory, hypothesis-generating investigation intended to identify potential genotype–phenotype relationships that warrant further validation in larger and independent cohorts.

### 3.2. Limitations

Several limitations of this study warrant consideration. First, the absence of an independent validation cohort limits the generalizability of our findings, as the observed associations were not confirmed in an external population. The eight variants analyzed were originally identified within the same cohort used for the present analyses (n = 48). Consequently, the current results are conditional on prior variant selection and cannot be regarded as an independent validation of genotype–phenotype associations. This design introduces the possibility of selection bias and “winner’s curse” inflation, whereby effect sizes observed in the discovery sample may be overestimated due to preferential selection of variants showing the strongest apparent associations. Therefore, the reported associations should be interpreted as exploratory and hypothesis-generating rather than confirmatory.

Furthermore, several observed genotype–phenotype relationships differed from expectations based on the original discovery analysis. These discrepancies may partly reflect the multifactorial nature of diabetic neuropathy, in which genetic susceptibility interacts with metabolic status, disease duration, environmental exposures, and potentially epigenetic regulatory mechanisms that may influence phenotypic expression. In addition, genotype–phenotype discordance may arise from incomplete penetrance, gene–environment interactions, or compensatory biological pathways.

Given the modest sample size (n = 48) and the small genotype subgroup counts, the statistical power to detect realistic genetic effects was limited, and the precision of several estimated associations is correspondingly low. The wide confidence intervals surrounding the odds ratio estimates indicate considerable uncertainty and potential instability of the estimated effect sizes. Consequently, the magnitude of the reported odds ratios should be interpreted with caution and should not be considered definitive measures of genetic effect. These findings are exploratory and require validation in larger independent cohorts. Furthermore, because genotype subgroup sizes differed across variants, test-specific power calculations for each SNP–phenotype comparison were not considered informative. Instead, a general detectable-effect-size analysis was performed to illustrate the statistical limitations imposed by the sample size. With approximately 24 individuals per group, the study had 80% power to detect only large effects (Cohen’s d ≈ 0.82) at a two-sided α = 0.05, while correlation analyses required effect sizes of approximately r ≥ 0.40 to achieve the same power. After correction for multiple testing, the detectable effects would be even larger. Therefore, the study was underpowered to detect small or moderate genotype–phenotype associations, and non-significant findings should not be interpreted as evidence of absence of association.

Another limitation relates to the unequal distribution of genotypes, particularly the low number of wild-type individuals, which may have reduced statistical power and introduced bias in group comparisons. As a consequence of the limited number of wild-type participants, wild-type and heterozygous genotypes were combined in the statistical analyses, which precluded the evaluation of specific inheritance models, including dominant and additive genetic effects. Furthermore, the low frequency of minor alleles may increase susceptibility to unstable estimates that are highly sensitive to outliers. In line with these limitations, the observed associations should be regarded as borderline significant, with q values ranging from 0.05 to 0.10, and may not persist in larger, independent datasets.

Regarding the bioinformatic limitations, although standard variant call format (VCF)-level quality-control procedures were applied during the original WES data processing workflow, some quality control (QC) parameters commonly reported in genetic association studies were not available for detailed variant-level reporting in the present genotype–phenotype analysis. These included mean sequencing depth, per-variant coverage metrics, Variant Quality Score Recalibration (VQSR) metrics, genotype quality distributions, and per-variant Hardy–Weinberg equilibrium test results specifically calculated in the control subgroup. Although Hardy–Weinberg equilibrium (HWE) deviation was included as part of the VCF-level SNP filtering workflow, control-specific HWE results were not recalculated separately for the present analysis. In addition, formal principal component analysis (PCA)-based ancestry verification was not performed. Therefore, residual population stratification cannot be fully excluded. Although the cohort was recruited from a clinically homogeneous Hungarian/European population and allele frequencies of the investigated variants were compared with European reference data, these measures cannot substitute for PCA-based ancestry assessment and do not completely eliminate the possibility of residual stratification. Consequently, both the absence of detailed variant-level QC reporting and the lack of formal ancestry verification should be considered when interpreting the exploratory genotype–phenotype associations reported in this study. Future studies in larger, independent cohorts should incorporate formal ancestry assessment together with comprehensive variant-level QC reporting to confirm the robustness, reproducibility, and generalizability of the observed associations.

Additionally, the applied allele dosage coding model (0–1–2) assumes a simple additive genetic effect, which may not fully capture the underlying biological relationships. Population heterogeneity and linkage disequilibrium with causal variants may also have contributed to the observed associations. Finally, some findings may partly reflect stochastic variation inherent to multiple testing.

Despite these limitations, the present results provide preliminary evidence supporting a potential relationship between selected genetic variants and neuropathy susceptibility. These findings may serve as a foundation for future studies designed to validate and further characterize the genetic contribution to neuropathy risk in larger and independent populations.

### 3.3. Future Work

The precise pathomechanism of diabetic neuropathy remains incompletely understood despite extensive research. Further investigation into the biological mechanisms underlying diabetic microvascular complications is essential to improve our understanding of disease development and progression. Studies aimed at identifying genetic contributors to neuropathy may provide valuable insight into individual susceptibility and the heterogeneity of clinical presentation.

The present study should be considered an exploratory, hypothesis-generating investigation. It intends to identify potentially relevant genetic variants associated with neuropathy-related clinical phenotypes. However, these preliminary findings require validation in substantially larger and independent patient cohorts to determine their reproducibility and clinical significance.

Experimental investigations using cellular or molecular models may help clarify whether these variants directly influence neuronal signaling, survival, apoptosis, or pathways implicated in diabetic neuropathy. In addition, gene-expression analyses could determine whether the investigated variants are associated with altered transcriptional activity of nearby or functionally related genes in relevant tissues.

Integration of expression quantitative trait locus (eQTL) analyses may further improve interpretation by identifying whether specific variants influence gene-expression levels in peripheral nerve tissue, blood, or other diabetes-relevant tissues. Such analyses could help establish mechanistic links between genetic variation and downstream molecular effects. Furthermore, longitudinal studies tracking neuropathy progression over time would be valuable to determine whether these variants are associated not only with neuropathy susceptibility but also with disease severity, progression rate, or long-term clinical outcomes.

## 4. Materials and Methods

### 4.1. Patient Selection

The study population comprised 48 individuals with type 2 diabetes, including 24 patients diagnosed with diabetic neuropathy (17 men and 7 women) and 24 patients without neuropathy (13 men and 11 women). Patients with neuropathy had a median age of 67.5 years (IQR: 63.5–71.0) compared with 58.0 years (IQR: 45.75–63.0) in patients without neuropathy (*p* = 0.0012). Median BMI was 31.5 kg/m^2^ (IQR: 26.9–34.45) in the neuropathy group and 29.25 kg/m^2^ (IQR: 27.03–31.6) in the non-neuropathy group (*p* = 0.1670). Median duration of diabetes was 11.5 years (IQR: 5.75–14.5) among patients with neuropathy and 8.0 years (IQR: 5.75–12.25) among those without neuropathy (*p* = 0.1322). Median HbA1c levels were 7.9% (IQR: 6.65–8.2) in the neuropathy group and 6.7% (IQR: 6.3–7.75) in the non-neuropathy group (*p* = 0.1376). The clinical cohort included in the present study is identical to that described in our previous publication [15].

Although the neuropathy group was older, the duration of diabetes did not differ significantly between groups, suggesting comparable long-term exposure to diabetes-related metabolic burden.

The study protocol was reviewed and approved by the local ethics committee (approval number 37596-8/2018/EÜIG). Written informed consent was obtained from all participants following appropriate explanation of the study procedures.

### 4.2. Neurological Assessment

A thorough neurological assessment was performed in all participants to exclude carpal tunnel syndrome and to identify clinical manifestations of neurological dysfunction. Special focus was placed on identifying muscle wasting and alterations in skin condition.

Cardiovascular autonomic neuropathy (CAN) was assessed using five standardized cardiovascular autonomic reflex tests. Parasympathetic function was evaluated through heart-rate responses to deep breathing (beat-to-beat variation), active standing (30:15 ratio), and the Valsalva maneuver (Valsalva ratio), whereas sympathetic function was assessed by blood pressure responses to standing. According to the Toronto Consensus Panel, the blood pressure response to sustained handgrip is no longer recommended as a routine clinical assessment and is considered primarily an investigational measure [32]. All measurements were obtained using a Cardiosys H-01 portable 12-lead electrocardiograph system (Experimetria Ltd., Budapest, Hungary). Cardiovascular autonomic neuropathy was defined by the presence of at least one abnormal or two borderline cardiovascular reflex test results. Test outcomes were graded as normal (0), borderline (1), or abnormal (2), enabling semi-quantitative assessment of neuropathy severity [33,34].

Sensory nerve function was assessed using the Neurometer^®^ CPT device (Neurotron Inc., Baltimore, MD, USA). Current perception thresholds (CPTs) were determined at the median and peroneal nerves using three stimulus frequencies (2000 Hz, 250 Hz, and 5 Hz), corresponding to the functional assessment of large myelinated (Aβ), small myelinated (Aδ), and unmyelinated (C) nerve fibers [33,35,36]. Reference values for CPT measurements were established according to previously published data by Evans et al. [37]. Sensory neuropathy was defined by the presence of at least two abnormal findings obtained by using either the Neurometer^®^ or the thermal sensory analyzer.

The normal values for the Neurometer^®^ and cardiovascular reflex tests are presented in Table 6 and Table 7.

Thermal sensory function was evaluated by measuring cold and heat detection thresholds using a Thermal Sensory Analyzer (TSA-II; Medoc Ltd., Ramat Yishai, Israel). Vibration perception threshold (VPT) was determined with a Vibratory Sensory Analyzer (VSA-3000) (Medoc Ltd., Ramat Yishai, Israel) on the same platform. Sensory nerve dysfunction was defined as the presence of at least two abnormal findings in either the upper or lower extremities at any tested frequency. The diagnosis of sensory neuropathy was established when at least two abnormal test results were present, fulfilling the predefined diagnostic criteria for sensory neuropathy.

The severity of neuropathic symptoms was assessed using the Neuropathy Total Symptom Score-6 (NTSS-6), a validated questionnaire that quantifies both the frequency and intensity of common symptoms of diabetic peripheral neuropathy, including shooting or lancinating pain, deep aching or tightness, allodynia or hyperalgesia, paresthesia, sensory loss, and burning sensations [38].

### 4.3. Genetic Analysis

We isolated genomic DNA from peripheral blood for the assay. This was performed using a Roche HighPure DNA Isolation Kit (Roche, Rotkreuz, Switzerland), according to the manufacturer’s instructions. The quantity of the isolated DNA was then analyzed using a Qubit dsDNA HS Assay Kit (Thermo Fisher Scientific, Waltham, MA, USA).

### 4.4. WES Data Processing

Whole-exome sequencing (WES) data were processed to extract variant information for patients categorized into two cohorts: neuropathic cases and non-neuropathic controls. Variant annotation was performed using ANNOVAR (08-06-2020 release), incorporating information from dbSNP, ClinVar, gnomAD, and OMIM databases. Sequencing reads were visualized using the Integrative Genomics Viewer (IGV 2.14.1), and duplicate reads were marked using Picard tools (2.27.5). Single-nucleotide polymorphism calling was performed on Variant Call Format files generated using the GATK pipeline, as described previously [14,15]. VCF files were merged using BCFtools (1.16), and the resulting variants were further annotated using SnpSift (5.1d). Variant annotation was based on the dbSNP reference database, hg38/build 151, obtained from the NCBI dbSNP repository.

Quality control of the raw VCF files was performed using PLINK v1.9. Predicted sample sex based on SNP data was compared with reported phenotypic sex to identify possible discrepancies. SNP filtering was performed using predefined thresholds for missingness rate (>0.05), minor allele frequency (MAF < 0.01), and Hardy–Weinberg equilibrium deviation (HWE *p* < 1 × 10^−10^), in accordance with PLINK v1.9 recommendations. Variants passing these QC steps were retained for downstream association analyses.

Association testing in the original WES analysis was performed in R v4.0.3 using the GENESIS Bioconductor package (2.28.0) to fit logistic regression models. Model estimates were adjusted for age, sex, and genetic relatedness. Genetic relatedness was estimated using a genetic relatedness matrix generated with the SNPRelate package (v1.32). In the present study, the previously identified neuropathy-associated variants were further evaluated in relation to clinical, neurophysiological, and laboratory parameters.

### 4.5. Bioinformatic and Statistical Methods

For clinical outcomes, patients were stratified by genotype into two groups: homozygous for the alternative allele (allele frequency (AF) = 1.0) and non-homozygous (wild-type or heterozygous, AF < 1.0). Due to the limited number of individuals carrying the wild-type genotype for several variants, a separate statistically meaningful wild-type group could not be established. Therefore, genotype stratification was performed by comparing homozygous carriers with a combined non-homozygous group, which included both wild-type and heterozygous individuals. Due to the small number of wild-type individuals in the study population, wild-type and heterozygous genotypes were combined for statistical analyses. Group differences were analyzed using Fisher’s exact test for binary variables and the Mann–Whitney U test for continuous clinical metrics. Given the limited sample size and potential deviation from normal distribution, empirical permutation-based *p*-values were additionally calculated for median differences to provide a more robust assessment of statistical significance. Permutation testing was performed using 100,000 iterations to reduce the influence of distributional assumptions and improve the reliability of observed associations.

Additionally, we assessed the relationship between allele dosage and neuropathy-associated measurements. Correlation analysis was performed using Spearman’s rank correlation. To account for multiple-hypothesis testing, *p*-values were adjusted using Storey’s q-value method [39]. Across the 8 investigated variants, 6 unique genotype patterns were identified and analyzed. Overall, 276 Spearman’s rank correlation analyses and 276 Mann–Whitney U tests were conducted across all phenotype measures.

To further assess the robustness of the observed associations and reduce the risk of false-positive findings in the context of multiple testing and limited sample size, permutation-based significance testing was additionally performed for all Spearman’s rank correlation analyses. Statistical significance was assessed using permutation-based *p*-values derived from 100,000 permutations.

Results were considered significant if the adjusted q-value was less than 0.05, and borderline (exploratory finding) if q-value was between 0.05 and 0.1. For multiple-testing correction, *p*-values obtained from the Mann–Whitney U tests and Fisher’s exact tests were combined into a single genotype–phenotype association testing family and adjusted jointly using Storey’s q-value method. Permutation testing for genotype-group comparisons was performed using the between-group median difference as the test statistic. Spearman’s rank correlation analyses were corrected separately because they addressed allele-dosage effects rather than genotype-group comparisons. Consequently, the results of the Mann–Whitney U test, median-difference permutation analysis and Spearman correlation analyses were interpreted independently, and statistical significance after FDR correction and permutation testing was assessed separately within each analysis.

To assess whether the observed genotype–phenotype associations remained independent of potential demographic confounders, multivariable linear regression analyses were performed with adjustment for age and sex. Statistical significance in multivariable linear regression analyses was defined as a two-sided *p*-value < 0.05.

Statistical analyses were performed using Python version 3.12 with the following libraries: statsmodels version 0.14.6, scikit-learn version 1.7.1, and SciPy version 1.16.0.

## 5. Conclusions

This exploratory study investigated the relationship between previously identified genetic variants and clinically relevant phenotypes of diabetic neuropathy in individuals with type 2 diabetes. Several associations were observed between selected variants and sensory neuropathy-related parameters. However, only a limited number of findings remained significant after correction for multiple testing and permutation analysis. Notably, the observed associations were predominantly restricted to sensory measures, while no consistent relationships were identified with autonomic parameters.

The partially inconsistent directionality of several genotype–phenotype relationships highlights the complex and potentially non-linear contribution of genetic factors to diabetic neuropathy. Given the small sample size, unequal genotype distribution, and lack of external validation, these findings should be interpreted cautiously. Nevertheless, the present results provide preliminary evidence that genetic variation may contribute to interindividual differences in neuropathy susceptibility and clinical manifestation. Further validation in larger, independent cohorts is required to confirm these associations and clarify their biological and clinical significance.

## Figures and Tables

**Table 1:** Demographic and clinical characteristics of the two study groups.

	T2DM with Neuropathy (*n* = 24)		T2DM Without Neuropathy (*n* = 24)		
	Median	IQR	Median	IQR	*p* Value
Age (years)	67.5	63.5–71	58	45.75–63	0.0012
Body mass (kg)	93	83.5–99	83.5	77.5–92.5	0.1009
Body height (cm)	172.5	165–180	169	163–176.75	0.4030
BMI (kg/m^2^)	31.5	26.9–34.45	29.25	27.025–31.6	0.1670
Systolic blood pressure (mmHg)	137.9	125.56–147.67	133.5	124.04–140.98	0.3842
Diastolic blood pressure (mmHg)	70.32	67.16–75.08	75.5	67.81–79.71	0.1718
Duration of diabetes (years)	11.5	5.75–14.5	8	5.75–12.25	0.1322
Sex (male/female)	17/7		13/11		0.371
Fasting blood sugar (mmol/L)	8.65	6.82–11.17	8.65	6.53–10.53	0.9912
HbA1c (%)	7.9	6.65–8.2	6.7	6.3–7.75	0.1376
Cholesterol (mmol/L)	4.45	4.1–5.73	5.05	4.13–5.78	0.5596
LDL cholesterol (mmol/L)	2.69	2.37–3.48	3.32	2.45–3.63	0.4480
HDL cholesterol (mmol/L)	1.23	1.01–1.32	1.19	0.92–1.34	0.6441
Triglyceride (mmol/L)	1.49	1.24–2.1	1.99	1.26–3.09	0.3065

Data are reported as median [IQR]. Sex distribution between groups was evaluated using Fisher’s exact test. Abbreviation: IQR, interquartile range.

**Table 2:** Whole-exome sequencing results.

Variant ID	Reference/ Alternative Allele	Position	Gene	Reference Allele Frequency (MAF) of European Population *	Logistic Regression Estimate (β)	Logistic Regression Estimate (β) Standard Error	OR for Reference Allele (95% CI)	*p* Value
rs922984	T/C	chr2:178751160 (GRCh38.p14)	*TTN*	0.070	3.248	0.989	26.69 (3.71–178.89)	0.001
rs2291313 rs4471922	T/C G/T	chr2:178767983 (GRCh38.p14) chr2:178768571 (GRCh38.p14)	*TTN*	0.202 0.205	2.304	0.738	22.65 (2.36–42.63)	0.002
rs6086563	C/G	chr20:8722498 (GRCh38.p14)	*PLCB1*	0.243	2.787	0.855	25.99 (3.04–86.87)	0.001
rs4241602	A/G	chr4:77066198 (GRCh38.p14)	*CCNI*	0.081	4.020	1.264	24.01 (4.67–664.5)	0.001
rs2396295 rs892204	A/G G/A	chr19:536437 (GRCh38.p14) chr19:536900 (GRCh38.p14)	*CDC34*	0.088 0.081	3.213	0.996	25.16 (3.53–175.07)	0.001
rs6682221	C/A	chr1:203305408 (GRCh38.p14)	*BTG2*	0.099	−2.761	0.893	0.045 (0.01–0.36)	0.002

The table demonstrates the chromosomal location and position of each genetic variant, alongside the associated minor allele frequencies, odds ratios (ORs), and corresponding 95% confidence interval (95% CI) for developing neuropathy. Odds ratios (ORs) were derived from logistic regression analyses performed in the discovery cohort and are not based on the current clinical correlation assessment. Pairwise linkage disequilibrium (LD) analysis indicated that rs2291313 and rs4471922 (*TTN*) are in complete LD (r^2^ = 1.00, D′ = 1.00), while rs2396295 and rs892204 (*CDC34*) are in near-complete LD (r^2^ = 0.985, D′ = 1.00), suggesting that each SNP pair likely represents a shared association signal rather than independent genetic effects.* European population allele frequencies gathered from the ALFA Allele Frequency Aggregator project [15,31].

**Table 3:** Associations between genetic variants and neuropathic symptoms, signs, and neurophysiological parameters.

SNP	Clinical Parameter	Median (Homozygous)	IQR	Median (Non-Homozygous)	IQR	*p*-Value	q-Value	Permutation q-Value
rs6682221	Heat detection threshold (left hand) (°C)	34.89 (n = 37)	34.005–36.57	33.45 (n = 11)	33.26–33.645	0.000150	0.02736	0.319858
	Heat detection threshold (right hand) (°C)	35.52 (n = 37)	34.67–37.74	33.98 (n = 11)	33.69–35.215	0.005115	0.097166	0.212697
	Heat detection threshold (left foot) (°C)	42.65 (n = 37)	38.38–46.18	37.69 (n = 11)	36.3–38.5	0.005780	0.097166	0.246164
	CPT (nervus peroneus) 2000 Hz (mm/s)	525 (n = 37)	428–717	378 (n = 11)	325–417	0.002849	0.097166	0.273679
	Cold detection threshold (right hand) (°C)	29.82 (n = 37)	27.61–30.44	30.88 (n = 11)	30.01–31.07	0.005551	0.097166	0.157689
rs4241602	CPT (nervus medianus) 250 Hz (mm/s)	120 (n = 42)	98.5–153.25	192.5 (n = 6)	175.25–212	0.002134	0.097166	0.148908
rs6086563	Cold detection threshold (right hand) (°C)	28.22 (n = 32)	25.93–29.52	24.81 (n = 16)	20–28.17	0.004122	0.097166	0.157649
rs2396295/rs892204	CPT (nervus medianus) 250 Hz (mm/s)	119 (n = 40)	97–154.25	173 (n = 8)	161.5–188.75	0.006162	0.097166	0.157649
	CPT (nervus medianus) 2000 Hz (mm/s)	356.5 (n = 40)	299.5–398	413.5 (n = 8)	406–426	0.006413	0.097166	0.279112

Data are reported as median [IQR]. Abbreviations: CPT, current perception threshold.

**Table 4:** Association between Neuropathy Total Symptom Score-6 (NTSS-6) and genetic variants (Fisher’s exact test).

SNP	Clinical Parameter	OR	*p*-Value	q-Value
rs2291313/rs4471922	NTSS-6	7.14	0.002949	0.097166
rs6086563	NTSS-6	7.14	0.004606	0.097166

Abbreviation: OR, odds ratio.

**Table 5:** The relationship between allele dosage and neuropathy-associated measurements (Spearman’s rank correlation, permutation correction).

SNP	Clinical Parameter	Correlation (r)	*p*-Value	q-Value	Permutation q-Value
rs6682221	Heat detection threshold (left hand)	0.552	0.000086	0.016	0.019
	Heat detection threshold (left foot)	0.411	0.0050	0.079	0.093
	Heat detection threshold (right hand)	0.400	0.0065	0.079	0.093
	Cold detection threshold (right hand)	−0.407	0.0055	0.079	0.093
	CPT nervus peroneus 2000 Hz	0.409	0.0039	0.079	0.090
	CPT nervus medianus 250 Hz	0.369	0.0099	0.090	0.099
rs2396295/rs892204	CPT nervus medianus 2000 Hz	−0.404	0.0044	0.079	0.090
	CPT nervus medianus 250 Hz	−0.399	0.0050	0.079	0.093
rs4241602	CPT nervus medianus 250 Hz	−0.452	0.0013	0.079	0.060
rs6086563	CPT nervus medianus 2000 Hz	−0.378	0.0081	0.086	0.094
	CPT nervus medianus 250 Hz	−0.38	0.0077	0.086	0.093
	CPT nervus peroneus 5 Hz	−0.373	0.0090	0.090	0.099
	Cold perception threshold (right hand)	−0.445	0.0022	0.079	0.090
	Cold perception threshold (right foot)	−0.405	0.0064	0.079	0.093

**Table 6:** Reference values for cardiovascular reflex tests [34].

Method	Tested Parameter	Normal Value	Borderline Value	Abnormal Value
Tests for the investigation of parasympathetic functions				
1. Deep breathing test	Beat-to-beat variation (beats/min)	≥15	11–14	≤10
2. Valsalva maneuver	Valsalva ratio	≥1.21	1.11–1.2	≤1.1
3. Heart rate response to standing	30/15 ratio	≥1.04	1.01–1.03	≤1.0
Tests for the investigation of sympathetic functions				
1. Blood pressure (BP) response to standing	Reduction in systolic BP (mmHg)	≤10	11–29	≥30
2. Handgrip test	Increase in diastolic BP (mmHg)	≥16	11–15	≤10

**Table 7:** Internationally accepted normal values with Neurometer device (note: 100 CPT units = 1 mA) [37].

Current Perception Threshold (Frequency)	Nervus Medianus (Normal Range in mm/s)	Nervus Peroneus (Normal Range in mm/s)
2000 Hz	120–398	179–523
250 Hz	22–189	44–208
5 Hz	16–101	18–170

## Data Availability

The original contributions presented in this study are included in the article/Supplementary Materials. Further inquiries can be directed to the corresponding author.

## Supplementary Materials

The following supporting information can be downloaded at: https://www.mdpi.com/article/10.3390/ijms27125487/s1.
