# Supplementary material for: Associations Between Previously Identified Genetic Variants and Clinical Phenotypes of Diabetic Neuropathy in Type 2 Diabetes: An Exploratory Analysis of the Discovery Cohort
Source: Int J Mol Sci. 2026 Jun 17;27(12):5487. doi: 10.3390/ijms27125487 (PMC13299346; doi:10.3390/ijms27125487)
Supplement: Supplementary file 1 [file ijms-27-05487-s001.zip › ijms-4357514-supplementary.pdf]

Supplementary Table S1. Results of Firth logistic regression analyses under the recessive genetic model for the association between investigated SNPs and neuropathy status. Models were adjusted for sex, age, and diabetes duration.

SNP	Adjusted OR (Recessive Model)	Adjusted p-value
rs922984	0.0133	0.000195
rs2291313	0.0742	0.000779
rs4471922	0.0742	0.000779
rs6086563	0.0721	0.001332
rs4241602	0.00922	0.000725
rs2396295	0.00586	0.000040
rs892204	0.00586	0.000040
rs6682221	11.6850	0.005457
